# Analysis of the interactive effects between sleep quality, trait mindfulness, vigor, and five types of negative emotions using EBICglasso network analysis

**DOI:** 10.3389/fpsyg.2025.1707960

**Published:** 2025-12-17

**Authors:** Xiaoqian Ding, Lijie Qiao, Qingmin Li, Zirong Yang

**Affiliations:** 1 College of Psychology, Liaoning Normal University, Dalian, China; 2 Department of Gastroenterology, Affiliated Zhongshan Hospital of Dalian University, Dalian, China

**Keywords:** EBICglasso network analysis, sleep quality, trait mindfulness, vigor, negative emotions

## Abstract

**Introduction:**

Network analysis offers a powerful approach for identifying complex interaction patterns that traditional statistical methods often overlook. However, limited research has applied network analysis to examine the interrelationships among sleep quality, trait mindfulness, vigor, and multiple negative emotions. This study aimed to investigate the structural associations among these psychological factors in Chinese college students.

**Methods:**

A total of 1,529 college students completed measures of sleep quality, trait mindfulness, vigor, depression, anxiety, confusion, hostility, and fatigue using the Pittsburgh Sleep Quality Index (PSQI), the Mindful Attention Awareness Scale (MAAS), and the Profile of Mood States (POMS). EBICglasso network analysis was conducted to estimate conditional associations and identify central nodes within the psychological network.

**Results:**

Results showed that Vigor-Activity was positively associated with Trait Mindfulness (*r* = 0.23) and negatively associated with Sleep Quality (*r* = –0.10). Depression-Dejection displayed the strongest edge with Anger-Hostility (*r* = 0.53) and was also positively associated with Tension-Anxiety (*r* = 0.36). Centrality analysis indicated that Depression-Dejection had the highest strength centrality, whereas Vigor-Activity demonstrated the highest betweenness and closeness centrality.

**Discussion:**

The findings suggest that depression functions as the core negative emotional factor within the network affecting sleep quality, while vigor serves as a key bridging variable linking trait mindfulness and sleep quality. These results support theoretical models of energy-related psychological functioning and highlight potential intervention targets for improving sleep quality among college students.

## Highlights

- Investigate the impact of negative emotions (depression, anxiety, confusion, hostility, and fatigue) on the sleep quality of college students, emphasizing the central role of depression in this network.
- Explore how trait mindfulness and vigor improve sleep quality through positive psychological factors, proposing that vigor may serve as a bridge between mindfulness and sleep quality.
- Utilize network analysis to study the interactions among sleep quality, mindfulness, vigor, and various negative emotions, revealing complex relationships and key psychological determinants.

## 1 Introduction

In China, approximately 25.7% of college students report experiencing sleep disturbances (Li et al., 2018). Sleep problems have a substantial negative impact on college students’ physical and psychological health, academic performance, and overall quality of life (Milojevich and Lukowski, 2016). Therefore, examining the psychological factors that influence sleep quality among college students is essential for developing effective intervention strategies.

Negative emotions are considered one of the primary psychological factors affecting sleep quality (Hoag et al., 2016). Emotional states such as anxiety, stress, depression, hostility, confusion, and fatigue can disrupt normal sleep patterns, leading to difficulties in sleep onset, nighttime awakenings, early morning awakenings, or an overall decline in sleep quality (Bouwmans et al., 2017; Brissette and Cohen, 2002; Caldwell et al., 2004; James and Gregg, 2004; Komarov et al., 2020). Negative emotions represent individuals’ psychological responses to adverse situations and encompass emotions such as anxiety, depression, hostility, confusion, and fatigue (Todaro et al., 2003). Empirical evidence suggests that interactions among negative emotions may deplete emotional resources and undermine coping capacity (Cramer et al., 2010; McElroy et al., 2018; Ruzzano et al., 2015), thereby increasing emotional instability, triggering internal conflict and social stress, and further facilitating the development of depressive symptoms (Price et al., 1994; Schofield et al., 2012). Depression, characterized by persistent sadness and loss of interest, has been shown to disrupt normal sleep architecture and reduce sleep quality (Becker et al., 2017). From a stress-response perspective, negative emotional states associated with depression may activate the hypothalamic–pituitary–adrenal (HPA) axis, elevating cortisol levels, reducing deep sleep, and impairing sleep structure (Herman et al., 2016). Depression may therefore be a core factor in the deterioration of sleep quality; however, due to methodological limitations, previous research has typically focused on the impact of single negative emotions on sleep quality, neglecting the multidimensional and complex nature of these influences (Ding et al., 2020). Consequently, the central role of depression in impairing sleep quality has not been fully recognized. In sum, more attention should be devoted to the impact of depression on sleep quality, as well as to the interactive effects between depression and other negative emotions. Accordingly, this study proposes Hypothesis H1: depression may occupy a central position within a psychological network composed of anxiety, depression, hostility, confusion, fatigue, and sleep quality, serving as the key negative emotional node influencing sleep quality.

Trait mindfulness and vigor are positive psychological factors that contribute to improvements in sleep quality (Bogusch et al., 2016; Ding et al., 2023). Howell et al. (2010) found that college students with higher levels of trait mindfulness typically report better sleep quality, including less daytime fatigue, fewer difficulties initiating sleep, and fewer sleep disturbances (Howell et al., 2010). A three-wave prospective study of working adults further demonstrated that vigor, as a positive emotional state, is associated with lower tendencies toward insomnia and exerts a protective effect on sleep quality (Armon et al., 2014). However, the internal mechanisms linking trait mindfulness, vigor, and sleep quality have not yet been systematically examined. Vigor reflects the extent to which individuals experience feelings of energy, activity, and mental alertness in their current emotional state (Berger and Motl, 2000). Trait mindfulness refers to an individual’s capacity to maintain awareness of and attention to present-moment experience and is regarded as a relatively stable dispositional characteristic (Brown and Ryan, 2003). Its core features involve sustained, nonjudgmental attention to ongoing experience (Brown and Ryan, 2003). Higher levels of trait mindfulness help individuals more accurately recognize their somatic and psychological states, enhance self-regulation and resource management, reduce cognitive rumination and automatic stress reactions, and mitigate psychological exhaustion (Bishop et al., 2004; Brown and Ryan, 2003), thereby facilitating the conservation and accumulation of psychological resources. According to Conservation of Resources theory, the accumulation of resources can trigger a “resource gain spiral,” promoting increases in positive emotions, subjective energy, and vigor (Hobfoll, 2001). Moreover, sleep is not merely a passive rest state but an active recovery process integrating neuroendocrine, immune, and emotion-regulation systems, playing a crucial role in maintaining physiological homeostasis and psychological adaptation (Harvey et al., 2011). In research on mindfulness and sleep, sleep quality is often conceptualized as a direct outcome of adaptive emotion regulation and stress-recovery processes (Ong et al., 2012). Shirom’s vigor model posits that vigor may function as a mediating construct linking individual characteristics to adaptive outcomes (Shirom, 2011). On the one hand, empirical findings indicate that trait mindfulness is positively associated with positive affect (Geschwind et al., 2011). The broaden-and-build theory of positive emotions proposes that positive emotions broaden individuals’ thought–action repertoires and facilitate the exploration of new ideas and possibilities, which is a direct experiential manifestation of vigor (Fredrickson, 2001). Consistent with this view, a study of working parents showed that individuals with higher trait mindfulness reported higher levels of vigor (Allen and Kiburz, 2012). On the other hand, vigor is closely linked to sleep quality. As a psychologically energetic state arising from positive emotions, vigor promotes higher levels of daytime activity (Lambert et al., 2011). Daytime activity provides necessary stimulation and energy expenditure for the body and brain, thereby optimizing nighttime rest and recovery and enhancing sleep quality (Isik and Ozer, 2022; Kobayashi et al., 1993). In turn, high-quality sleep further enhances individuals’ daytime vigor (Schmitt et al., 2017), forming a positive reciprocal cycle. Additionally, studies have shown that college students with higher trait mindfulness tend to have better sleep quality (Bogusch et al., 2016). Taken together, individuals with higher trait mindfulness are more likely to experience elevated vigor and better sleep quality. Therefore, this study proposes Hypothesis H2: vigor may function as a “bridging” mechanism between trait mindfulness and sleep quality, linking positive psychological resources with restorative functioning and serving as an important mediating node in the promotion of sleep quality.

Previous studies have typically employed traditional linear methods to examine the relationships among sleep quality, trait mindfulness, vigor, and single negative emotions separately (Ding et al., 2020). However, to date, no study has systematically investigated the conditional dependencies and interactions among sleep quality, trait mindfulness, vigor, and multiple negative emotions from a unified network-system perspective. Traditional linear analytic approaches tend to focus on single pathways or direct effects between variables and are limited in their ability to capture multiple potential associations and conditional dependency structures. In essence, such approaches reduce complex psychological phenomena to independent linear relations, overlooking holistic network characteristics and mutual influences among variables and failing to adequately reflect the structural properties of psychological systems (Kwon and Cho, 2007). Network analysis, a modern statistical technique grounded in graph theory, has been widely applied in psychology and related disciplines (Cramer et al., 2010; McElroy et al., 2018; Zhao et al., 2023). From a network perspective, the interactions among variables may provide more parsimonious and informative explanations for the emergence and maintenance of social phenomena and symptom constellations (Borsboom et al., 2021). By examining the pattern of nodes and edges and utilizing visualization techniques, network analysis can reveal latent, non-intuitive associations and structural patterns among variables (Epskamp et al., 2018). This approach overcomes key limitations of traditional linear models by identifying those factors that are most central for sleep quality and by clarifying conditional dependency relations among variables (Epskamp et al., 2018). In the present study, we adopted the EBICglasso (Extended Bayesian Information Criterion Graphical LASSO) model. This method is based on Gaussian Graphical Models (GGMs) and uses LASSO regularization to obtain sparse network estimates, preserving key edges while suppressing noisy connections (Epskamp et al., 2018; Friedman et al., 2008). Compared with traditional latent-variable approaches such as structural equation modeling (SEM) or causal graphical models such as Bayesian networks, EBICglasso does not require predefining latent structures or directional pathways. Instead, it directly estimates partial correlations that represent conditional independence relations among observed variables, making it particularly suitable for exploring complex associative structures among psychological constructs (Epskamp et al., 2018; Friedman et al., 2008). Furthermore, by incorporating the Extended Bayesian Information Criterion (EBIC) penalty term, EBICglasso achieves an appropriate balance between model fit and complexity, helping to avoid overfitting and enhancing the robustness and replicability of the estimated network (Foygel and Drton, 2010). Within network analysis, centrality indices are used to characterize the structural position and functional role of each psychological variable in the overall system (Opsahl et al., 2010). Strength centrality reflects the sum of edge weights connected to a node and indicates its degree of connectedness and overall influence; in psychological networks, nodes with high strength are typically regarded as structural “core” components with broad connections and impact (Opsahl et al., 2010). Closeness centrality quantifies the average shortest path from a node to all other nodes; higher closeness suggests greater accessibility and integrative efficiency within pathways of psychological information processing and regulation (Opsahl et al., 2010). Betweenness centrality captures how frequently a node lies on the shortest paths between other node pairs; higher betweenness indicates that a node serves as a “bridge” connecting different psychological modules and plays a key role in linking subcomponents of the system (Opsahl et al., 2010).

In summary, network analysis offers a powerful framework for understanding and exploring the complex relationships among psychological variables. Therefore, the present study applied network analysis to examine a joint network of sleep quality, trait mindfulness, vigor, anxiety, depression, hostility, confusion, and fatigue. Our goals were to uncover the structural relations among these variables, identify nodes that play central or bridging roles in the network, and explore how trait mindfulness and vigor jointly contribute to sleep quality at the system level.

## 2 Methods

### 2.1 Participants

Participants were selected using a simple random sampling method from multiple provinces in China, including Beijing, Liaoning, Sichuan, and Guangxi. A total of 1,529 college students participated in the survey. After excluding participants with incomplete responses, 1,374 participants provided usable data. The final sample consisted of 27.2% males (*n* = 374) and 72.8% females (*n* = 1,000), with an average age of 18.49 years (SD = 0.50).

### 2.2 Measures

#### 2.2.1 Mindful attention awareness scale (MAAS)

This scale primarily assesses the degree of attention and awareness an individual has towards the present moment. The MAAS was originally developed by Brown and Ryan (2003), and the present study used the validated Chinese version of the scale. The MAAS is a unidimensional structure comprising 15 items. Participants are required to select the description that best fits their actual situation over the past week (including the day of the survey) on a scale from 1 (“almost never”) to 6 (“almost always”). Higher scores indicate a higher level of present-moment awareness and attention in daily life. The internal consistency coefficient of the scale in this study was 0.86.

#### 2.2.2 Pittsburgh sleep quality index (PSQI)

This scale consists of 19 items and includes seven factors: subjective sleep quality, sleep latency, sleep duration, habitual sleep efficiency, sleep disturbances, use of sleeping medication, and daytime dysfunction. Each factor is scored on a scale from 0 to 3, and the scores of the seven factors are summed to obtain the total PSQI score. Higher scores indicate poorer sleep quality. In this study, the internal consistency coefficient of the scale was 0.88.

#### 2.2.3 Profile of mood states (POMS)

This scale consists of 65 adjectives describing different emotional states and is used to evaluate and understand the mood of the respondents. Each item is rated on a scale from 0 (“not at all”) to 4 (“extremely”). The 65 emotional states are categorized into six dimensions through factor analysis: Tension-Anxiety, Depression-Dejection, Anger-Hostility, Vigor-Activity, Fatigue-Inertia, and Confusion-Bewilderment. Except for Vigor-Activity, the dimensions mainly represent negative emotional states, with higher scores indicating worse emotional states. In this study, the internal consistency coefficient of the scale was 0.92.

## 3 Data analyses

This study used SPSS 19.0 for descriptive statistics and correlation analysis. Network analysis was conducted using R 3.6.0 statistical software. First, the network structure was constructed using the least absolute shrinkage and selection operator (LASSO) in the R package qgraph (version 2.2.1), Then, centrality indices (strength centrality, closeness centrality, and betweenness centrality) were calculated to quantify the importance of each node in the network structure (Opsahl et al., 2010). Finally, the stability of the centrality indices was quantified using the R package bootnet (version 1.5.6) by reducing the sample size in the network structure. The stability of the centrality indices was assessed by calculating the stability coefficient. A stability coefficient greater than 0.25 is considered acceptable, while a coefficient greater than 0.5 indicates good stability (Epskamp et al., 2018).

## 4 Results

### 4.1 EBICglasso network analysis

#### 4.1.1 Estimation of the overall network

**Figure 1:**
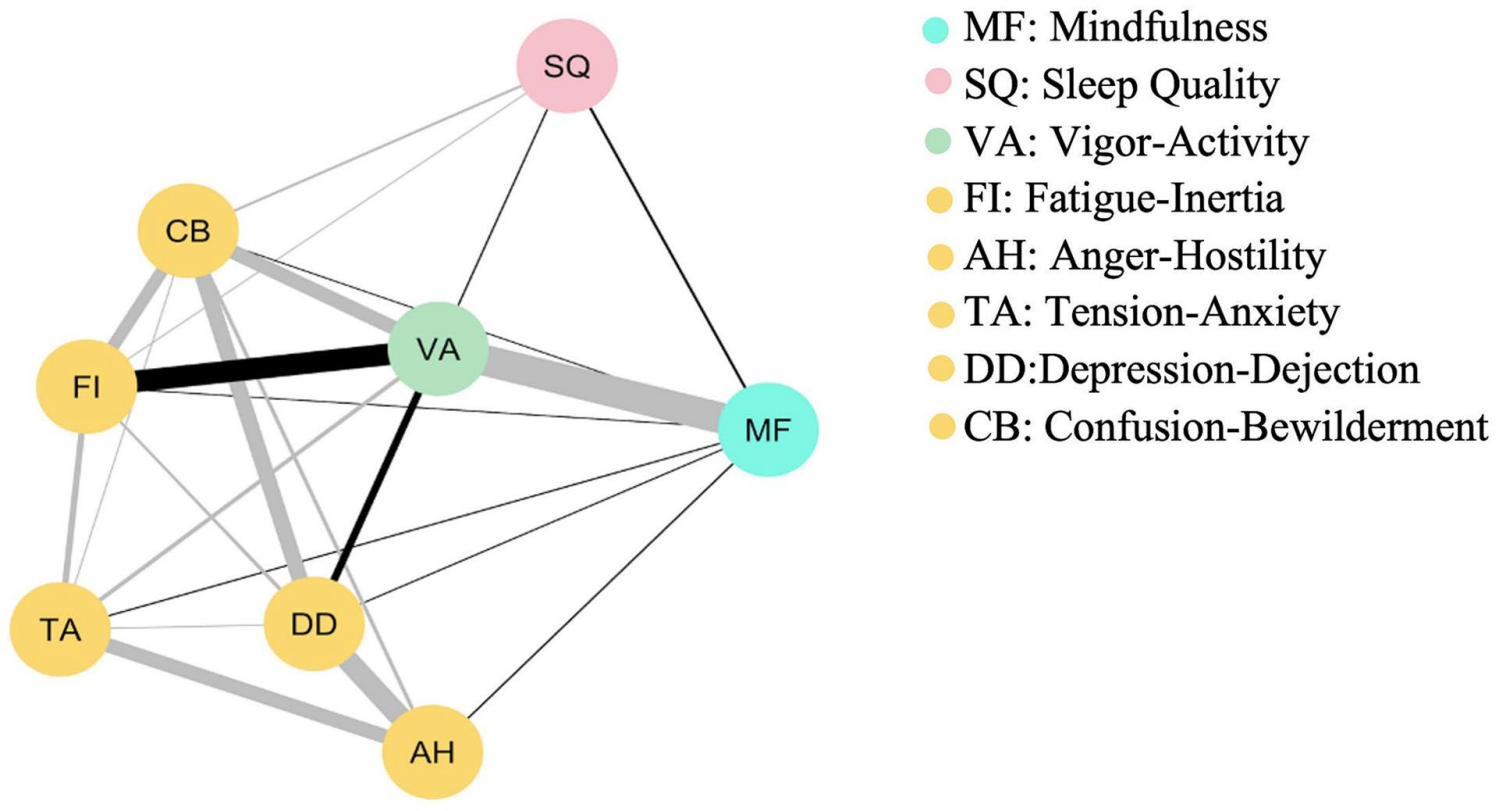
Network analysis diagram illustrating the relationships between trait mindfulness, sleep quality, and emotional states. Gray Solid lines represent positive correlations, while black solid lines indicate negative correlations. The wider the line is, the larger the correlation coefficient is.

The EBICglasso network of Sleep Quality, Trait Mindfulness, Vigor-Activity, Depression-Dejection, Tension-Anxiety, Confusion-Bewilderment, Anger-Hostility, and Fatigue-Inertia among 1,374 Chinese college students is shown in Figure 1. This network consists of 8 nodes and 26 non-zero edges.

Table 1 presents the adjacency matrix of Sleep Quality, Trait Mindfulness, Vigor-Activity, Depression-Dejection, Tension-Anxiety, Confusion-Bewilderment, Anger-Hostility, and Fatigue-Inertia.

**Table 1:** Adjacency matrix of all variables.

Variables	TA	DD	AH	VA	FI	CB	MF	SQ
TA	--							
DD	0.36	--						
AH	0.15	0.53	--					
VA	0.11	−0.21	0.00	--				
FI	0.12	0.17	0.16	−0.23	--			
CB	0.16	0.11	0.05	0.31	0.41	--		
MF	−0.04	−0.05	−0.02	0.23	−0.03	−0.10	--	
SQ	0.01	0.03	0.00	−0.10	0.05	0.05	−0.03	--

MF = Mindfulness; SQ = Sleep Quality; VA = Vigor-Activity; FI = Fatigue-Inertia; AH = Anger-Hostility; TA = Tension-Anxiety; DD = Depression-Dejection; CB = Confusion-Bewilderment.

Trait mindfulness was directly associated with Vigor-Activity (*r* = 0.23) and Confusion-Bewilderment (*r* = −0.10). Additionally, it had direct but weaker associations with Tension-Anxiety, Depression-Dejection, Anger-Hostility, and Fatigue-Inertia. Trait mindfulness was negatively correlated with sleep quality (*r* = −0.03).

Depression-Dejection showed the strongest edge strength with Anger-Hostility (*r* = 0.53) and was also positively correlated with Tension-Anxiety (*r* = 0.36). Vigor-Activity had negative correlations with Depression-Dejection (*r* = −0.21) and Fatigue-Inertia (*r* = −0.23), and positive correlations with Confusion-Bewilderment (*r* = 0.31) and Tension-Anxiety (*r* = 0.11). There was a direct negative correlation between Vigor-Activity and sleep quality (*r* = −0.10). Fatigue-Inertia and Confusion-Bewilderment also had direct associations with sleep quality, though these were relatively weaker.

#### 4.1.2 The estimation of centrality and its stability

**Figure 2:**
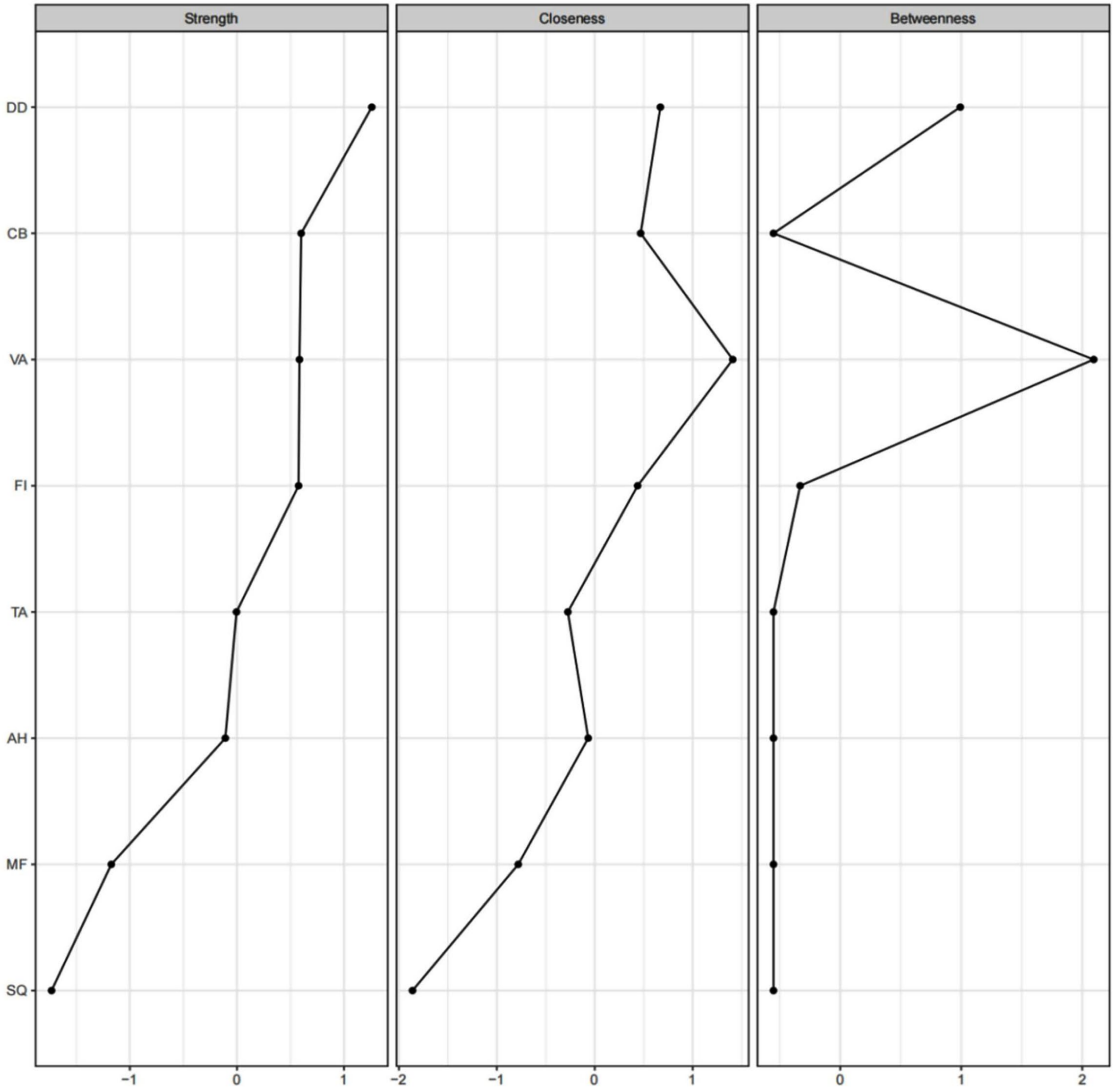
Centralization measurement of network nodes. The figure illustrates the centrality indices of different nodes, including strength, closeness, and betweenness centrality. The horizontal axis represents standardized *z*-scores, while the vertical axis displays the node labels. The nodes are ordered by Strength centrality from highest to lowest, providing a clear representation of the importance of each node within the network.

**Figure 3:**
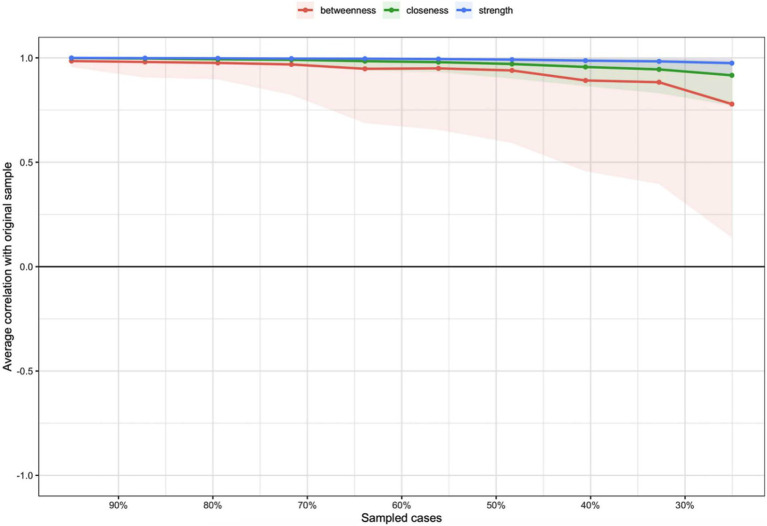
Stability analysis of centrality metrics. The graph illustrates the stability of three centrality measures (strength, closeness, and betweenness) as cases are progressively removed from the sample. The *x*-axis represents the percentage of the original sample retained, while the *y*-axis shows the average correlation of the centrality measures with the original sample. Shaded areas indicate confidence intervals, and different colored lines represent the three centrality measures.

The network’s centrality measures indicated that Depression-Dejection had the highest strength centrality, whereas Vigor-Activity exhibited the highest betweenness and closeness centralities (Figure 2). The stability coefficients for strength, betweenness, and closeness centrality in the overall network model were 0.75, 0.60, and 0.75, respectively (Figure 3). According to Epskamp et al. (2018), a centrality stability (CS) coefficient above 0.70 signifies the maximum acceptable reduction in sample size, while a CS coefficient above 0.50 is deemed acceptable (Epskamp et al., 2018).

## 5 Discussion

This study aimed to examine the psychological factors influencing sleep quality among college students by applying EBICglasso network analysis to uncover the structural associations among sleep quality, trait mindfulness, vigor, depression, anxiety, confusion, hostility, and fatigue. Based on the computation of centrality indices, the study identified the relative importance of these variables within the psychological system from a graph-theoretical perspective. The results showed that, within the overall psychological network, depression exhibited the highest strength centrality, whereas vigor demonstrated the highest betweenness and closeness centrality.

The present study found that depression was significantly and positively associated with both anxiety and hostility, and it showed the strongest edge weights among all variable pairs. Within the overall network structure, the depression node exhibited the highest strength centrality. According to graph-theoretical principles, high strength centrality indicates that a node is connected to a larger number of strong edges, thereby occupying a more prominent structural core position in the network (Epskamp et al., 2018; Opsahl et al., 2010). This suggests that depression is not only strongly linked to multiple negative emotional states but also shows broad conditional dependencies with sleep quality, making it the most integrative and influential node in the network. This finding is consistent with previous evidence showing that depression is highly co-occurring with other negative emotions within affective networks (McElroy et al., 2018). Prior research has demonstrated substantial overlap among depression, anxiety, hostility, and related negative emotions in terms of affective experience, cognitive processing, and emotion regulation mechanisms. Moreover, research in affective neuroscience and sleep science indicates that depression and sleep quality are interconnected through multiple bidirectional pathways, including shared neurotransmitter systems (e.g., serotonin, norepinephrine), impaired emotion-regulation mechanisms, and disruptions in circadian rhythm functioning (Herman et al., 2016). These mechanisms may contribute to the mutually reinforcing cycle between depression and sleep disturbance. Although these mechanisms cannot be construed as causal pathways in the present study, they offer conceptual explanations for why depression showed the strongest structural centrality in the network model. The prominent structural role of depression within the psychological network underscores its importance as a key negative emotional factor influencing sleep quality. This finding enhances our understanding of how depression may serve as a nexus linking emotional dysfunction with sleep disturbances and highlights the need for psychological interventions to prioritize alleviating depressive symptoms to improve sleep quality and overall mental health. Future research may further investigate whether improvements in depression lead to enhanced sleep quality through longitudinal or experimental designs and assess the effectiveness of different intervention strategies in reducing depression and its associated sleep problems.

Furthermore, the present study found that vigor demonstrated the highest betweenness and closeness centrality in the network. Within a graph-theoretical framework, higher betweenness centrality indicates that a node plays a more critical “path-bridging” role between different psychological variables, whereas higher closeness centrality reflects shorter average distances to all other nodes, suggesting greater accessibility and integrative efficiency within the overall psychological system (Epskamp et al., 2018; Opsahl et al., 2010). Based on these properties, vigor exhibited a clear structural bridging function, linking the psychological subsystems involving trait mindfulness and sleep quality. This structural characteristic aligns with Shirom’s vigor model, which conceptualizes vigor as an energetic and positive psychological resource that connects individual characteristics with adaptive outcomes (Shirom, 2011). The current findings indicate that the association between trait mindfulness and sleep quality at the system level is embedded in a shared linkage pattern that depends on vigor. From a psychological-mechanism perspective, trait mindfulness and vigor are closely related. Previous research has shown that trait mindfulness can reduce cognitive rumination, enhance emotional regulation efficiency, and lower psychological burden (Brown and Ryan, 2003; Robins et al., 2012), thereby facilitating the accumulation of positive psychological resources. According to Conservation of Resources (COR) theory, resource accumulation generates a self-reinforcing “resource gain spiral” that promotes increases in energy, motivation, and vigor-related experiences (Hobfoll, 2001). Thus, individuals with higher levels of trait mindfulness tend to exhibit higher levels of vigor—an association supported by existing empirical evidence (Allen and Kiburz, 2012). Vigor is also closely tied to sleep quality. Individuals with higher vigor typically engage in more active daytime behaviors, experience more effective physical and psychological relaxation, maintain more stable emotional and physiological rhythms, and ultimately achieve better sleep quality (Du et al., 2020; Hartescu et al., 2015). Therefore, vigor may function as the psychological energy conduit through which trait mindfulness and sleep quality are jointly connected, making it a key bridging node within the network structure identified in this study.

In sum, as a malleable psychological capacity (Kiken et al., 2015), trait mindfulness may influence sleep quality in part through its shared structural linkages with vigor. The findings of this study offer implications for future interventions: enhancing vigor—such as through mindfulness training or positive activity interventions—may represent an effective pathway for improving sleep quality. Future research may benefit from employing longitudinal or experimental designs to examine the temporal dynamics through which vigor operates as a bridging mechanism between trait mindfulness and sleep quality, thereby deepening our understanding of the functional role of this structural bridge within the psychological system.

By applying network analysis, the present study simultaneously examined sleep quality, trait mindfulness, vigor, depression, anxiety, confusion, hostility, and fatigue within a unified psychological system. The results identified the core role of depression and the bridging role of vigor. These findings not only support the vigor model but also extend the Conservation of Resources theory, enriching prior research in this domain. By revealing a more complex pattern of interrelationships among psychological variables than is captured by traditional linear approaches, the current study provides important theoretical and practical insights for developing more targeted psychological interventions and strategies to improve sleep quality.

### 5.1 Limitations and future directions

This study has several limitations. First, the data were collected using a cross-sectional design, which prevents the identification of causal relationships among variables. Future research may consider employing randomized controlled trials (RCTs) or longitudinal designs to examine the dynamic mechanisms and causal pathways underlying factors that influence sleep quality over time. Second, all variables in this study were assessed through self-report measures, which may be subject to subjective biases. Future studies should incorporate more objective assessments (e.g., polysomnography, electroencephalography) to obtain more accurate and reliable indicators of sleep and psychological functioning. Third, the sample consisted of Chinese university students whose cultural background and demographic characteristics are relatively homogeneous, which may limit the cross-cultural generalizability of the findings. Trait mindfulness, vigor, and certain emotional variables may be shaped by culturally specific norms and patterns of expression, and cultural factors may influence the conditional dependencies within the network structure. Future research would benefit from conducting multicultural or cross-cultural multigroup network analyses to examine the stability of the EBICglasso-derived network across different cultural contexts and to further explore how cultural and social contextual factors operate within psychological networks. Finally, the conclusions of this study are based on a healthy university student sample with limited representativeness. Future work should expand the target population to more diverse groups (e.g., older adults, adolescents) in order to further assess the generalizability and external validity of the findings.

## Data Availability

The raw data supporting the conclusions of this article will be made available by the authors, without undue reservation.
